# Radioguided Localisation Techniques for Non-Palpable Breast Lesions: An Umbrella Review

**DOI:** 10.3390/jcm15020750

**Published:** 2026-01-16

**Authors:** Marco Cuzzocrea, Cesare Michele Iacovitti, Nickolas Peradze, Maria Luisa Gasparri, Simone Schiaffino, Lorenzo Rossi, Gaetano Paone, Giorgio Treglia

**Affiliations:** 1 Division of Nuclear Medicine, Ente Ospedaliero Cantonale (EOC), 6500 Bellinzona, Switzerland; marco.cuzzocrea@eoc.ch (M.C.); cesaremichele.iacovitti@eoc.ch (C.M.I.); gaetano.paone@eoc.ch (G.P.); 2 Department of Gynecology and Obstetrics, Ente Ospedaliero Cantonale (EOC), 6900 Lugano, Switzerland; nickolas.peradze@eoc.ch (N.P.); marialuisa.gasparri@eoc.ch (M.L.G.); 3 Faculty of Biomedicine, Università della Svizzera Italiana (USI), 6900 Lugano, Switzerland; simone.schiaffino@eoc.ch; 4 Department of Radiology, Ente Ospedaliero Cantonale (EOC), 6900 Lugano, Switzerland; 5 Centro di Senologia della Svizzera Italiana, Istituto Oncologico della Svizzera Italiana (IOSI), Ente Ospedaliero Cantonale (EOC), 6962 Lugano, Switzerland; lorenzo.rossi@eoc.ch; 6 Faculty of Biology and Medicine, University of Lausanne, 1015 Lausanne, Switzerland

**Keywords:** breast-conserving surgery (BCS), radioguided occult lesion localisation, ROLL, sentinel node and occult lesion localisation, SNOLL, wire-guided localisation, WGL

## Abstract

**Background:** Accurate localisation of non-palpable breast lesions is essential for the optimization of breast-conserving surgery (BCS) outcomes. While wire-guided localisation (WGL) remains widely used, radioguided techniques—including Radioguided Occult Lesion Localisation (ROLL) and Radioactive Seed Localisation (RSL)—have been proposed to improve margin clearance, reduce reoperations, and enhance patient outcomes. This umbrella review aimed to critically appraise and synthesize evidence from systematic reviews and meta-analyses on radioguided localisation techniques for non-palpable breast lesions, with a primary focus on comparison with wire-guided localisation (WGL). **Methods:** A comprehensive literature search was conducted using PubMed/Medline and the Cochrane Library databases for eligible systematic reviews/meta-analyses published until 2024, focusing on outcomes such as relative efficacy, safety, margin positivity, re-excision rates, operative efficiency, and patient-related outcomes. **Results:** In total, 35 records were retrieved, but only 10 evidence-based articles were selected. Radioguided approaches achieved high localisation success (often exceeding 95%) and fewer positive margins compared to WGL, while reoperation findings were mixed. Operative/localisation times were generally shorter for radioguided methods, with comparable specimen volume/weight and favourable safety profiles. **Conclusions:** Radioguided localisation methods provide superior or at least equivalent outcomes compared with WGL and can improve workflow; Sentinel Node and Occult Lesion Localisation (SNOLL) may support combined lesion localisation and sentinel node evaluation. Further high-quality, standardized comparative studies are needed to define the optimal resection ratio, protocol standardization and cost of the radioguided techniques and other newer probe-guided methods.

## 1. Introduction

The management of non-palpable breast lesions represents one of the most important challenges in breast-conserving surgery (BCS), a widely accepted therapeutic modality for early-stage breast cancer [1]. Since the advent of mammography screening programmes, the early detection of non-palpable lesions has increased significantly [2]. It is estimated that up to 25–33% of breast cancer diagnoses involve non-palpable lesions, which require precise localisation techniques to ensure complete excision and minimize the risk of recurrence [3,4].

Compared to mastectomy, BCS offers a significant advantage in terms of esthetic results and quality of life without compromising effectiveness in terms of oncological outcomes [5]. However, the success of this approach largely depends on the accurate localisation of the lesions during surgery, in order to remove the tumour with clear margins and to preserve as much healthy tissue as possible for an optimal aesthetic result.

Wire-guided localisation (WGL), introduced in the 1960s, has been the standard technique for decades [6]. It involves the insertion of a wire into the breast under radiological guidance to mark the position of the lesion. Although widely available and inexpensive, this technique has several limitations, such as the displacement of the wire during transport or surgery [7,8,9,10], which compromises the accuracy of localisation, the patient’s perceived pain and discomfort [11], and the difficulty of performing an accurate resection, which often results in positive tumour margins, necessitating surgical reinterventions [12].

Over the past two decades, several wire-free techniques have been developed to overcome the limitations of WGL, including Radioguided Occult Lesion Localisation (ROLL) [13], Radioactive Seed Localisation (RSL) [14], and Ultrasound-Guided Surgery (UGS) [15]. ROLL was introduced in the 1990s; it uses a radioactive tracer to locate lesions. RSL is a method that uses radioactive seeds of 125-I to mark lesions, allowing for greater operational flexibility. UGS allows real-time intraoperative localisation, eliminating the need for radioactive materials and offering greater adaptability during surgery.

In addition, new techniques, such as intraoperative supine magnetic resonance imaging (SMRI) [16], Anchor-Guided Localisation (AGL) [17], Cryo-Assisted Localisation (CAL) [18], indocyanine green fluorescence-guided localisation (IL) [19], magnetic marker localisation (ML) [20,21], radiofrequency-guided localisation (RGL) [22], and radar reflectors (RR) [23], have emerged, which promise to further expand the landscape of available options. However, evidence supporting their systematic adoption remains limited due to the small number of comparative studies. The international prospective cohort trial MELODY is currently the largest ongoing registry with the aim of assessing the most adopted breast localisation techniques and devices from several perspectives [24].

This umbrella review aims to critically appraise and synthesize evidence from systematic reviews and meta-analyses on radioguided localisation techniques for non-palpable breast lesions, with a primary focus on comparing radioguided approaches, i.e., ROLL and RSL, to the traditional WGL. Their relative efficacy, safety, margin positivity, re-excision rates, operative efficiency, and patient-related outcomes were evaluated via analysis.

## 2. Materials and Methods

This umbrella review was reported in accordance with the “Preferred Reporting Items for Systematic Reviews and Meta-Analyses” (PRISMA 2020 statement) as the guiding reporting framework. The review protocol was not registered (consistent with PRISMA checklist item 24).

As a first step, a comprehensive literature search was conducted using PubMed/Medline and the Cochrane Library databases, identifying systematic reviews and meta-analyses published on the selected topic until 2024. The selected search string was ((radioguided) OR (occult lesion) OR (ROLL) OR (SNOLL)) AND (breast) AND ((systematic review) OR (meta-analysis)).

We included systematic reviews and/or meta-analyses that (i) evaluated radioguided localisation techniques for non-palpable breast lesions in the setting of breast-conserving surgery, including Radioguided Occult Lesion Localisation (ROLL), Sentinel Node and Occult Lesion Localisation (SNOLL), and other radioguided approaches (RSL) compared with wire-guided localisation (WGL) or other localisation methods; (ii) reported at least one pre-specified outcome of interest (localisation success/accuracy, margin status/positive margins, re-excision and/or reoperation rates, localisation time and/or operative time, specimen volume/weight, sentinel lymph node identification, perioperative complications, patient-reported satisfaction/pain, and/or cosmetic outcomes); and (iii) provided sufficient information to allow qualitative synthesis of findings (and quantitative results when available).

We excluded (i) primary studies (randomized trials, cohort studies, case–control studies), case reports/series, editorials, letters, narrative or non-systematic reviews; (ii) articles not addressing radioguided localisation for non-palpable breast lesions; (iii) reviews focusing solely on non-radioguided techniques; and (iv) studies not reporting any of the outcomes of interest.

## 3. Results

**Figure 1:**
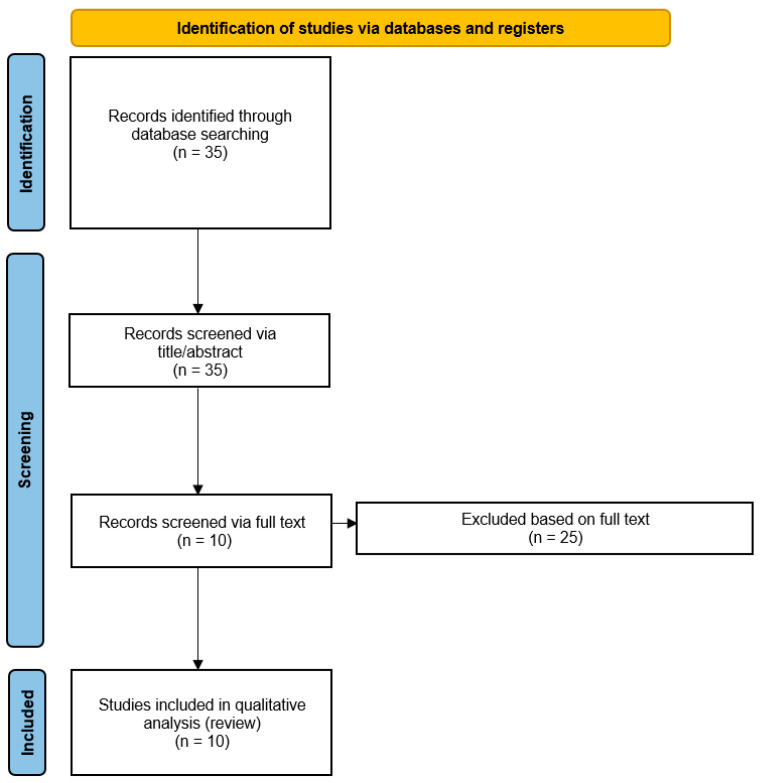
PRISMA flowchart summarizing the study selection process. PRISMA: Preferred Reporting Items for Systematic Reviews and Meta-Analyses.

From the comprehensive literature search using the PubMed/Medline and Cochrane Library databases, 35 records were retrieved, but only 10 evidence-based articles (systematic reviews and/or meta-analyses) were selected based on the target research question [25,26,27,28,29,30,31,32,33,34], according to the predefined inclusion and exclusion criteria. The main characteristics of the selected systematic reviews and/or meta-analyses are presented in Table 1 and summarized below. The selection process is summarized in Figure 1.

The ten selected evidence-based articles were published in the last two decades, from 2008 to 2024. Across the included systematic reviews and meta-analyses, all localisation techniques demonstrated high accuracy in identifying non-palpable breast lesions. Radioguided approaches consistently achieved reliable intraoperative detection, with studies reporting success rates exceeding 95%, comparable or superior to WGL [26,27,33]. In particular, a review on the combination of ROLL and sentinel lymph node localisation (SNOLL) confirmed almost perfect lesion detection, with success ranging from 95.5% to 100% [28]. None of the network meta-analyses formally pooled localisation accuracy as an outcome, but two confirmed broadly equivalent reliability across all tested methods [31,32].

When considering surgical margin status, the majority of reviews agreed that radioguided methods reduce the risk of positive margins compared to WGL. Lovrics and colleagues [26] reported a significant advantage for ROLL/RSL, with a pooled odds ratio of 0.367 (95% CI 0.277–0.487), while Kiruparan et al. [33] observed similar results favouring ROLL (OR 0.60, 95% CI 0.44–0.97). Sajid et al. (2012) [27] also described lower margin positivity with ROLL in a pooled analysis of randomized trials. In contrast, the Cochrane review [29] found only a non-significant trend toward improved margins (RR: 0.74; 95% CI: 0.42–1.29). More recent analyses provided further nuance: Moreira et al. [30] concluded that radioguided approaches were superior to WGL, with a tendency for RSL to outperform ROLL, whereas Ferreira et al. [34] demonstrated that RSL and ROLL were clearly superior to WGL, with no significant differences between the two radioguided approaches.

Reoperation rates followed a similar pattern. Lovrics et al. [26] showed significantly fewer reinterventions with radioguided localisation compared to WGL (OR: 0.347; 95% CI: 0.250–0.481), although other analyses reported more mixed results. Both Sajid et al. and the Cochrane review [27,29] described a non-significant reduction with ROLL, while Kiruparan et al. [33] found no difference. Ferreira et al. [34] concluded that RSL was superior to WGL and comparable to ROLL.

Efficiency outcomes favoured radioguided techniques. Sajid et al. [27] showed that both localisation and operative times were significantly shorter with ROLL, with mean differences of −6.09 (*p* < 0.00001) and −5.33 (*p* < 0.00001) minutes, respectively, compared to WGL. Kiruparan et al. [33] likewise reported localisation −5.83 min (*p* = 0.0003) and operative −1.95 min (*p* = 0.02) with ROLL vs. WGL. Athanasiou et al. [31] identified a modest reduction in operative time with UGS in pairwise comparisons, although this did not remain significant in network analysis, while Davey et al. [32] observed no consistent differences across methods. Altogether, the evidence indicates that ROLL improves workflow efficiency over WGL.

Specimen volume and weight were consistently reported as comparable across techniques, with no significant differences between WGL, ROLL, and RSL [27,29,32,33].

In terms of sentinel node identification, only SNOLL specifically addressed this outcome, demonstrating high identification rates between 88.2% and 100% [28]. None of the other reviews evaluated SLNB success systematically.

Patient-reported outcomes were sparsely investigated. The Cochrane review [29] noted limited and heterogeneous data on cosmetic results, without significant pooled findings. Davey et al. [32] reported improved satisfaction with magnetic marker localisation compared to WGL, but evidence remains insufficient for firm conclusions.

Safety outcomes were generally favourable across all methods, with no significant differences in perioperative complication rates reported by Sajid et al. [27], Chan et al. [29], and Kiruparan et al. [33]. Ferreira et al. [34] confirmed the equivalent safety of RSL compared to both WGL and ROLL. The only complication unique to WGL was wire migration. The main results of the umbrella review are summarized in Table 2.

## 4. Discussion

This umbrella review provides a comprehensive synthesis of systematic reviews and meta-analyses evaluating radioguided localisation techniques for non-palpable breast cancer, focusing on comparing radioguided approaches (ROLL and RSL) to the traditional WGL. The accumulated evidence highlights the progressive evolution from traditional WGL toward more accurate and effective methods, with radioguided techniques demonstrating clear advantages in several surgical outcomes.

The findings across multiple reviews confirm that all approaches achieve high localisation accuracy, with success rates exceeding 95% in most series. WGL remains widely used, yet its technical limitations—particularly the potential for wire displacement—can negatively impact surgical control, leading the surgeon to adopt other more reliable techniques [35]. ROLL and RSL consistently provide equivalent or superior lesion detection. Although accuracy per se is uniformly high, the distinction between these methods becomes more evident when margin status and reoperation rates are considered.

Positive margins remain a critical outcome influencing the need for re-excision, patient morbidity, and long-term oncological safety. Three meta-analyses [26,27,33] demonstrated significant reductions in positive margins with ROLL compared to WGL, while a Cochrane review [29] reported only a non-significant trend, likely reflecting heterogeneity, small study sizes, and mixed technique groupings. Moreira et al. [30] and Ferreira et al. [34] highlighted the potential superiority of RSL, showing that it consistently outperforms WGL and is at least equivalent to ROLL. Collectively, the balance of evidence indicates that ROLL improves the likelihood of negative margins compared with WGL, with a less consistent impact on re-excision once broader variability in case-mix and definitions is considered [27,29,33]. This consistent finding, despite limited trial numbers, signals a potential paradigm shift in localisation practice.

Reoperation rates mirror the results observed for margin status. While earlier systematic reviews suggested that radioguided localisation reduces reintervention rates [26], subsequent analyses were less conclusive, with several reporting no significant differences [27,29,33]. However, both Moreira et al. [30] and Ferreira et al. [34] indicated that RSL may lower the reoperation rate compared to WGL.

In terms of efficiency, ROLL consistently reduced both localisation and operative times compared to WGL, a finding supported by multiple meta-analyses [27,33]. In contrast, Davey et al. [32] reported no consistent differences across methods. In general, these results suggest that radioguided techniques may optimize operative workflow. Importantly, specimen volume and weight were consistently similar across all methods, indicating that differences in margin positivity and reoperation are not attributable to excision size but rather to more accurate targeting.

Safety outcomes are reassuring, with all localisation techniques demonstrating low complication rates. WGL carries the unique risk of wire migration, which may compromise lesion targeting, while radioguided methods do not. RSL and ROLL were found to be at least as safe as WGL, and RSL offers additional logistical benefits due to more flexible scheduling compared to radiotracer-based methods. Patient satisfaction and cosmetic outcomes, while increasingly important in breast-conserving surgery remain underreported and inconsistently measured.

This umbrella review synthesizes the highest evidence of systematic reviews and meta-analyses, primarily focused on providing a structured comparison between ROLL/RSL and WGL strategies. Nevertheless, several limitations warrant consideration. First, the certainty of our conclusions is constrained by the quality and heterogeneity of the included reviews: definitions of “positive margin” varied across studies, perioperative endpoints were not uniformly reported, and patient-reported outcomes were inconsistently captured. Second, we lacked access to patient-level data, precluding adjustment for key effect modifiers (e.g., DCIS vs. invasive histology, radiologic phenotype, neoadjuvant therapy, centre/surgeon experience). Third, temporal and technological drift (e.g., improvements in imaging, probes, and surgical techniques) across inclusion periods likely contributes to between-study heterogeneity and may attenuate pooled effects when translated to present-day practice. Fourth, publication bias and selective outcome reporting could not be ruled out at the umbrella level because small-study effects and funnel-plot assessments were inconsistently evaluated in the source reviews. These constraints advise for cautious interpretation of pooled effects, particularly for re-excisions and patient-centred outcomes, while the convergent signal on margin status and operative efficiency in favour of radioguided techniques—especially ROLL versus WGL—remains comparatively robust.

Finally, for some comparisons (e.g., RSL vs. ROLL), the articles included report qualitative or narrative conclusions without providing numerical data that can be extracted for tabulation. Therefore, Table 1 only shows comparisons with extractable values. However, the manuscript discusses the qualitative indications present in the original articles.

## 5. Conclusions

The collective evidence from systematic reviews and meta-analyses suggests that radioguided localisation methods provide superior or at least equivalent outcomes compared with wire-guided localisation. ROLL and RSL reduce positive margins and streamline operative workflow. SNOLL provides a unique dual benefit by combining lesion localisation with sentinel lymph node biopsy. Altogether, the data support a gradual but definitive shift away from WGL toward more effective alternatives. Although the current evidence strongly advocates clinical adoption, addressing logistical considerations, training requirements, and resource availability is necessary. Future research should focus on the optimal resection ratio, protocol standardization, and cost of the radioguided techniques and other newer probe-guided methods.

## Figures and Tables

**Table 1:** Summary of systematic reviews and meta-analyses included in the umbrella review.

Authors (Year)	[Ref.]	Studies; *n*. of Patients	Localisation Rate (95% CI)	Positive Margins (95% CI)	Reoperation (95% CI)	Localisation/Operative Time (MD, *p*)	SLNB Success (95% CI)	I^2^	Publication Bias
van der Ploeg et al. (2008)	[25]	5 comparative (ROLL *n* = 263 vs. WGL *n* = 253) + 4 describe ROLL + SN	Tracer correctly placed 95–99%	Negative margins ROLL 69–84% vs. WGL 44–60% (NR)	NR	Faster with ROLL: US ROLL 6 min vs. WGL 15 min; stereotactic ROLL 12 min vs. 20 min (both *p* < 0.001); another trial ROLL 16 min vs. WGL 23 min (*p* = 0.058)	ROLL + SN identification 90–100%	NR	NR
Lovrics et al. (2011)	[26]	27 studies (10 pooled; 4 RCTs + 6 cohorts); *n* = 1379	NR	OR 0.367 (95% CI 0.277–0.487), *p* < 0.001	OR 0.347 (95% CI 0.250–0.481), *p* < 0.001	Radiologist localisation faster in 4 studies; pooled op time NS (*p* = 0.053)	NR	Moderate–high	Not assessed
Sajid et al. (2012)	[27]	4 RCTs; *n* = 449 (ROLL *n* = 218; WGL *n* = 231)	Comparable	OR 0.47 (95% CI 0.22–0.99), *p* < 0.05	No significant difference (trend ↓ ROLL)	Localisation (MD −6.09 min, *p* < 0.00001); Operative (MD −5.33 min, *p* < 0.00001)	NR	Low (I^2^ < 30%)	Not evaluated
Ahmed et al. (2013)	[28]	7 studies; *n* = 983	95.5–100%	NR	2–12%	One study: ROLL 32.7 min vs. WGL 36.5 min (NS)	88.2–100%	NR	NR
Chan et al. (2015)	[29]	11 RCTs; *n* = 1273 (6 RCT ROLL vs. WGL)	Localisation failure RR 0.60 (95% CI 0.16–2.28), (NS)	RR 0.74 (95% CI 0.42–1.29), (NS)	RR 0.51 (95% CI 0.21–1.23), (NS)	NR	NR	Variable	No evidence found
Moreira et al. (2020)	[30]	49 studies (ROLL/RSL vs. WGL) (NR subset)	NR	ROLL vs. WGL RR 0.72 (95% CI 0.57–0.91), *p* = 0.007	ROLL vs. WGL RR 0.70 (95% CI 0.56–0.88), *p* = 0.002	Localisation (MD −9.39, *p* = 0.2); Operative (MD −3.05 min, *p* = 0.006)	NR	NR	NR
Athanasiou et al. (2022)	[31]	18 RCTs; *n* = 3112	NR	ROLL vs. WGL (NS)	NR	NR	NR	NR	NR
Davey et al. (2022)	[32]	24 RCTs; *n* = 4225 (WGL *n* = 2045; ROLL *n* = 640)	NR	ROLL vs. WGL OR 0.664 (95% CI 0.394–1.03), (NS)	ROLL vs. WGL OR 0.715 (95% CI 0.347–1.33), (NS)	ROLL vs. WGL OR −2.85 min (95% CI −8.18–2.23), (NS)	NR	NR	Low to moderate risk of bias
Kiruparan et al. (2022)	[33]	9 RCTs; *n* = 1096 (WGL *n* = 534; ROLL *n* = 562)	OR 1.34 (95% CI 0.40–4.53), (NS)	OR 0.60 (95% CI 0.44–0.97), *p* = 0.03	OR 1.42 (95% CI 0.83–2.43), *p* = 0.20 (NS)	Localisation (MD −5.83 min, *p* = 0.0003); Operative (MD −1.95 min, *p* = 0.02)	NR	Low–moderate	No asymmetry
Ferreira et al. (2024)	[34]	46 studies (4 studies RSL vs. ROLL, *n* = 1550; 43 studies RSL vs. WGL, *n* = 19,820)	NR	RSL vs. WGL RR 0.78 (95% CI 0.70–0.87), *p* < 0.001	RSL vs. WGL RR 0.71 (95% CI 0.61–0.84), *p* < 0.001	NR	NR	NR	NR

**Legend**: ROLL = Radioguided Occult Lesion Localisation; RSL = Radioactive Seed Localisation; WGL = wire-guided localisation; US = Ultrasound; SLNB = Sentinel Lymph Node Biopsy; RCT = Randomized Controlled Trial; OR = odds ratio; RR = Risk Ratio; MD = mean difference; CI = Confidence Interval; NR = Not Reported; NS = not significant; I^2^ = measure of heterogeneity; ↓ = reduction.

**Table 2:** Summary of the main results of the umbrella review. Rows represent the intervention groups and columns represent the comparison groups.

	WGL				ROLL				RSL			
	LR	PM	Re-Op	LT	LR	PM	Re-Op	LT	LR	PM	Re-Op	LT
**WGL**					**=/** **−**	**−**	**=/** **−**	**−**	**=/** **−**	**−**	**=/** **−**	
**ROLL**	**=/** **+**	**+**	**=/** **+**	**+**						**=**		
**RSL**	**=/** **+**	**+**	**=/** **+**			**=**						

**Legend**: LR = localisation rate; LT = localisation/operative time; PM = positive margins; Re-Op = reoperation; ROLL = Radioguided Occult Lesion Localisation; RSL = Radioactive Seed Localisation; WGL = wire-guided localisation; ‘+’ row better than column; ‘−’ row worse than column; ‘=’ no difference (green/red used only as visual aid).

## Data Availability

The data presented in this study are available on request from the corresponding author.
